# The Potentials of Melatonin in the Prevention and Treatment of Bacterial Meningitis Disease

**DOI:** 10.3390/molecules26051419

**Published:** 2021-03-05

**Authors:** Dong Zhang, Shu Xu, Yiting Wang, Guoqiang Zhu

**Affiliations:** 1 College of Veterinary Medicine, Yangzhou University, Yangzhou 225009, China; d150089@yzu.edu.cn (D.Z.); MX120170707@yzu.edu.cn (S.X.); MX120180700@yzu.edu.cn (Y.W.); 2 Jiangsu Co-Innovation Center for Prevention and Control of Important Animal Infectious Diseases and Zoonoses, Yangzhou 225009, China

**Keywords:** bacterial meningitis, neuron injury, melatonin, neuroprotection

## Abstract

Bacterial meningitis (BM) is an acute infectious central nervous system (CNS) disease worldwide, occurring with 50% of the survivors left with a long-term serious sequela. Acute bacterial meningitis is more prevalent in resource-poor than resource-rich areas. The pathogenesis of BM involves complex mechanisms that are related to bacterial survival and multiplication in the bloodstream, increased permeability of blood–brain barrier (BBB), oxidative stress, and excessive inflammatory response in CNS. Considering drug-resistant bacteria increases the difficulty of meningitis treatment and the vaccine also has been limited to several serotypes, and the morbidity rate of BM still is very high. With recent development in neurology, there is promising progress for drug supplements of effectively preventing and treating BM. Several in vivo and in vitro studies have elaborated on understanding the significant mechanism of melatonin on BM. Melatonin is mainly secreted in the pineal gland and can cross the BBB. Melatonin and its metabolite have been reported as effective antioxidants and anti-inflammation, which are potentially useful as prevention and treatment therapy of BM. In bacterial meningitis, melatonin can play multiple protection effects in BM through various mechanisms, including immune response, antibacterial ability, the protection of BBB integrity, free radical scavenging, anti-inflammation, signaling pathways, and gut microbiome. This manuscript summarizes the major neuroprotective mechanisms of melatonin and explores the potential prevention and treatment approaches aimed at reducing morbidity and alleviating nerve injury of BM.

## 1. Introduction

Bacterial meningitis (BM) is the major cause of the central nervous system (CNS) infectious diseases among infants, adults, and older people, which usually induce high mortality and 50% of the survivors left permanent neurological sequelae [1,2]. Bacterial meningitis can affect anyone of any age, including neonatal bacterial meningitis, adult bacterial meningitis, and senile bacterial meningitis. Simultaneously, environmental conditions and immunocompromised people are susceptible to bacterial meningitis. The pathogenesis of bacterial meningitis is as follows: firstly, meningitis bacteria can colonize the skin or different mucosal surfaces of healthy persons, then disseminated by blood and penetrated host barrier, finally resulting in systemic infection and neuronal injury [3,4]. The pathogenesis of BM mainly includes high-level bacteremia in the bloodstream, the destruction of the BBB integrity, and cerebrospinal fluid (CSF) pleocytosis, overwhelming inflammatory response in the CNS [3,5,6], which results in serious damage to the nervous system and even death. Up to now, lots of reports have shown the molecular mechanisms of BM resulting from bacterial ligand–receptor interactions, degradation of tight junction proteins, high matrix metalloproteinases (MMPs) expression, oxidative stress, and associated signaling pathways. Although antibiotics and vaccines have been able to significantly reduce meningitis mortality for clearing bacteria, the emergence of drug-resistance bacteria and the limitations of vaccine serotype make BM still cause high morbidity and seriously neurological damage sequelae. Hence, new therapies of prevention or treatment need to improve the BM.

Melatonin is a hormone with various biological functions. It is first found to be secreted by the pineal gland and then melatonin can be found to be secreted by other various organs including skin, retina, kidneys, pancreas, ovaries, and gastrointestinal tract [7,8,9]. Melatonin with amphiphilicity can easily cross the BBB so that it enters the central nervous system (CNS) and the cerebrospinal fluid (CSF) [7]. This is particularly important for the effective prevention and treatment of CNS diseases after the supplement of exogenous melatonin. Initially, melatonin is well known for regulating circadian rhythms, sleep, and reproduction [10,11]. Subsequently, a number of studies have shown that melatonin has many other crucial functions, such as antibacterial, antioxidant, anti-inflammation, anti-apoptosis regulating the immune system, and gut microbiome [12,13,14,15]. At present, the beneficial effects of melatonin on protecting the BBB integrity, inhibiting neuronal and glial injury in various models of CNS disease have been well documented [16,17,18,19]. In addition, the levels of metabolism productions of melatonin in the CSF, *N*^1^-acetyl-*N*^2^-formyl-5-methoxykynuramine (AFMK), and *N*^1^-acetyl-5-methoxykynuramine (AMK) were elevated, and then it was found that they exerted neurocyte-protective properties in the conditions of inflammation and oxidative stress, which also play a critical role in anti-inflammatory and neuroprotection in the CNS [20].

Hence, this review mainly focuses on the neuroprotective effects of melatonin, which include antibacterial, blocking the interaction of bacteria and receptors, protection of the BBB integrity, resisting oxidative stress, anti-inflammatory activity, and major signaling pathway in both in vivo and in vitro models.

## 2. Bacterial Meningitis

### 2.1. Epidemiological Characteristics

Bacterial meningitis is one of the top ten causes of infectious-disease death, and there are approximately 1.2 million bacterial meningitis cases per year worldwide, 300,000 of which are estimated fatal in 2015 [21,22]. Meanwhile, permanent neurological sequelae occur in half of the survivors [1,23]. The occurrence of bacterial meningitis is affected by many elements, such as geographic location, socioeconomic status, seasonal variations, age, vaccination, and health status of the individual [24]. In developing countries, the morbidity of meningitis is significantly higher than in developed countries [2,25,26]. In sub-Saharan Africa, bacterial meningitis can reach 1000 cases per 100,000 people per year, whereas the incidence is 1–2 cases per 100,000 people per year in the UK [27]. Epidemiological surveys published in 2018 show that the incidence of bacterial meningitis in Western countries (Finland, Netherlands, the US, and Australia) gradually declined to 0.7–11 per 100,000 in the past 10–20 years, and in African countries (Burkina Faso and Malawi), bacterial meningitis can still reach 10–40 per 100,000 persons per year [22]. This finding demonstrates the bacterial meningitis is closely related to environmental and economic conditions.

Bacterial meningitis can affect anyone of any age, but different bacteria mainly infect the hosts of different ages. For example, Group B *Streptococcus* (GBS) and *Escherichia coli* K1 are mainly meningitis pathogens of the neonate and infant [3,28,29] and *Streptococcus pneumonia* and *Neisseria meningitides* mainly infect adults [3,30,31,32]. Simultaneously, immunocompromised neonate or infant and adult with digestive tract diseases, smoking, drinking, human immunodeficiency virus (HIV), or cancer are susceptible to bacterial meningitis. Most bacterial meningitis remain an acute and severe disease with a high risk of complications that lead to death or permanent sequelae. These complications include shock, respiratory failure, organ failure, intracranial complication stroke or seizures, etc. [33,34,35]. Among elderly patients with bacterial meningitis, septicemia and respiratory failure were the primary cause of death; the main complication among younger patients was brain herniation [36]. If the host survives post-infection, it may leave pathogen-specific sequelae, such as deafness, blindness, or certain kinds of retardation.

### 2.2. Pathogenesis of Bacterial Meningitis

**Figure 1:**
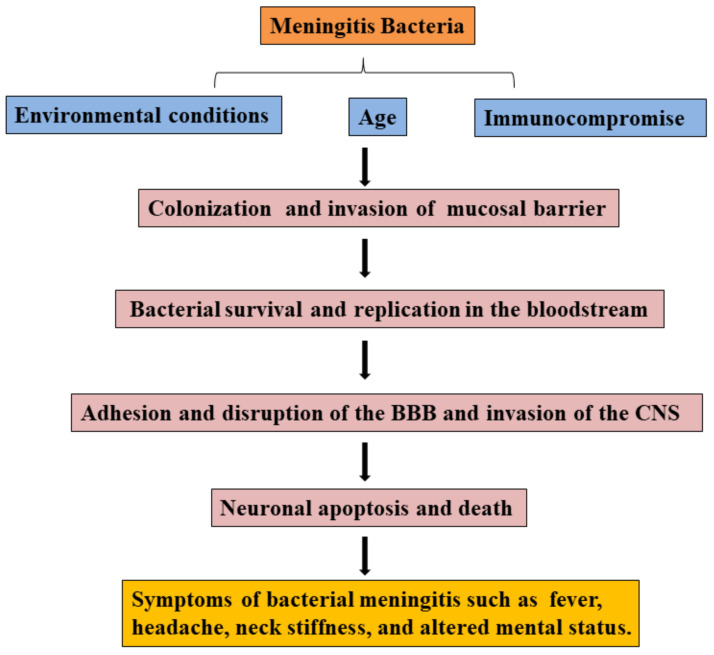
Overview of the pathogenic process in bacterial meningitis.

Most pathogens of bacterial meningitis firstly colonize oropharynx, nasopharynx, or digestive mucosal surfaces and cross the mucosal barrier, survive and disseminate in the bloodstream, then adhere and invade the BBB, eventually invade into the CNS (see Figure 1) [3]. Meningitis bacterium, including *Streptococcus pneumonia*, *Neisseria meningitis*, *Group B Streptococcus*, *Streptococcus aureus*, or *Escherichia coli K*1, can colonize mucosal surfaces of healthy people. Pathogens cross the mucosal barrier into the bloodstream, and bacterial survival and replication in the bloodstream are the prerequisites for reaching the BBB [3]. In bacterial meningitis, the complement system and Toll-like receptors (TLR) play an important role in clearing pathogens. For example, complement factors were induced to deposit on the surface of pathogens for promoting phagocytosis of phagocytes [37], and TLR activation prevents bacterial growth by inducing inflammation [38,39,40].

Bacteria initially adhere and invade the BBB via the interaction between bacterial components and host receptors, then degrade tight junction proteins of BBB depending on bacterial products or other components for invading into the CNS. Meanwhile, lots of pro-inflammatory cytokines, Matrix metalloproteinases (MMPs) expression, and free radicals contribute to the BBB disruption. Immune cells, including recruited neutrophils and resident cells in the CNS, can be activated by bacteria and express more pro-inflammatory cytokines, MMPs, and free radicals. Following invasion into the CNS, bacteria cause neurocyte and neural injury, which is usually caused by bacterial products, excessive inflammatory response, and major signaling pathways. In recent years, reports have found that the gut microbiome is one of the considerable factors in patients with bacterial meningitis, and gut disorders contribute to the development of meningitis.

In the early stages of bacterial meningitis, antibiotics are regarded as an important therapy to increase survival and reduce morbidity. The principal treatment strategy is to clear bacteria and reduce CNS damage. However, most of the antibiotics do not efficiently cross the BBB and play an antibacterial role in the CNS. Moreover, investigators explore novel therapeutic approaches for improving the experimental meningitis models by modulating reactive nitrogen species (RNS), inhibiting caspase or inflammatory factors, coagulant, or complement cascades [41,42,43]. For vaccines, researchers are interested in developing the efficacy of polysaccharide conjugates without serotype replacement or with broad and ideally universal coverage for different bacterial meningitis. Table 1 gives an overview of treatments available to prevent meningitis bacteria and cure patients. Moreover, the released production of bacteria can still stimulate immune system response, promote neutrophil invasion, and activate resident immune cells in CNS, resulting in death or severe sequelae of nerve damage. Therefore, bacterial meningitis would benefit from new therapies and more effective drugs, which can prevent or cure the disease and alleviate nerve damage for reducing morbidity, mortality, and sequela.

## 3. Melatonin

### 3.1. The Chemical and Physical Characteristic of Melatonin

Melatonin is a tryptophan derivative, belongs to indole heterocyclic compounds. Chemically, it is *N*-acetyl-5-methoxytryptamine, also called pineal hormone. The molecular formula of melatonin is C_13_H_16_N_2_O_2_ and its molecular weight is 232.28. Meanwhile, melatonin also has fat solubility and water solubility, which can effectively enter the cells and cross the BBB. In vertebrates, the secretion of melatonin has an obvious circadian rhythm, which is inhibited during the day (0–20 pg/mL) and active (60–200 pg/mL) at night. The secretion rate of melatonin is about 29 mg/day in humans.

### 3.2. The Synthesis and Metabolism of Melatonin

It was firstly found that melatonin was produced from pinealocytes in the pineal gland, and then it was later discovered that melatonin is also synthesized in other organs, in which the content of melatonin secreted from the gut is two orders of magnitude greater than that in the pineal gland. The processes of melatonin biosynthesis include hydroxylation, decarboxylation, acetylation, and methylation. Tryptophan as an initial precursor is turned into 5-hydrpxytryptophan by tryptophan hydroxylase. Then, 5-hydroxytryptophan decarboxylase decarboxylate 5-hydrotryptophan into 5-hydroxytryptamine (also called serotonin). Next, serotonin is acetylated into *N*-acetylserotonin via serotonin *N*-acetyl transferase. Finally, *N*-acetyserotonin is methylated to *N*-acetyl-5-methoxytryptamine [68].

Compared to melatonin synthesis, melatonin metabolism has multiple pathways which are complex with various enzymatic, pseudoenzymatic, and free radical interactive processes [69]. At present, the productions of melatonin metabolism mainly include 6-Hydroxymelatonin, 2-Hydroxymelatonin, cyclic 3-hydroxymelatonin, AFMK, and AMK, which play an important role under the condition of oxide stress [20]. During enzymatic processes, cytochrome P450 can catabolize melatonin to 6-hydroxymelatonin and then is conjugated to sulfate to form 6-hydroxymelatonin sulfate in the cerebral cortex, kidney, and heart of rats. In addition to enzymatic processes, melatonin can interact with ONOO^−^, ^·^OH or under the condition of UV-B irradiation to form 6-hydroxymelatonin. 6-Hydroxymelatonin can inhibit lipoperoxidation and the production of ROS, resulting in decreasing neurotoxicity. 2-Hydroxymelatonin is the production of melatonin which interacts with ROS/RNS, and it was also found that UV-B irradiation can induce melatonin to form 2-hydroxymelatonin in the cells. Cyclic 3-hydroxymelatonin is an oxidative melatonin metabolite and a reliable biomarker of endogenous ^·^OH levels. It was also reported that the interaction of melatonin with ONOO^−^ promoted Cyclic 3-hydroxymelatonin formation. Meanwhile, cyclic 3-hydroxymelatonin can form AFMK by scavenging radicals. The coexistence of cyclic 3-hydroxymelatonin and AFMK was usually found in the metabolic pathway of melatonin both in vitro or in vivo. For the past years, scholars were becoming more and more interested in AFMK [70]. It was found that AFMK was a pivotal molecule and original production in melatonin metabolism. It was initially found that AFMK formed via indoleamine 2,3-dioxygenase catalyzed melatonin, then it was found that AEMK is produced by an interaction of melatonin with H_2_O_2_. Subsequently, it was reported that UV irradiation can induce melatonin to form AFMK. In addition, AFMK can be further deformylated into AMK via arylamineformamidase, hemoperoxidases, or interacted with ROS/RNS. Many studies demonstrate that organisms can produce AFMK, including unicellular alga, metazoans, plants rodents, and humans. Meanwhile, AFMK and AMK may be exclusive metabolites of melatonin in tissues, especially in CNS. For example, the concentration of AFMK was greatly high (13,200 pg/mL) in the CSF of patients with meningitis, which was three orders of magnitude higher than healthy persons [71]. Leukocytes are another critical site for producing AFMK. The levels of AFMK were significantly increased in activated leukocytes. In cellular organelles, mitochondria are the major site for forming AFMK. Cytochrome C in mitochondria can catalyze melatonin into AFMK.

### 3.3. The Bioavailability of Melatonin

Melatonin as a health product is widely sold in the market. Over the past few decades, it was found that the bioavailability of melatonin in humans was significantly lower than that in rodents. Melatonin bioavailability is affected by various factors in humans, such as sexual difference, the heterogenic properties of cytochrome C P450 subtype gene expression, and the interactions with drugs. At present, the commercially available formula for melatonin is a 3 mg tablet. This dose is beneficial to promote sleep for some subjects, yet may not be effective to relieve insomnia and other related disorders in others. For example, the bioavailability of melatonin in females is 16.8 ± 12.7%, and that in males is 8.6 ± 3.9% following oral administration [72]. Fourtillan and colleagues have shown that the plasma level of melatonin was 165 pg/mL in males and 200 pg/mL in females after intravenous administration [72]. However, the level of melatonin dropped to 70 pg/mL in males and females after 1 h, which indicated over the physiological level and were eliminated by the liver. These results suggested that sex and route of administration affected the bioavailability of melatonin in the host. However, intranasal administration is not suitable for clinical application because of strong irritation. Mao et al. improved intranasal administration that developed melatonin starch microspheres [73]. The absorption of melatonin is increased and the bioavailability is markedly improved, but it disrupts the circadian rhythms of patients.

Later, researchers have used melatonin in combination with other drugs to increase the bioavailability of melatonin in humans. In healthy subjects, co-administration of melatonin with fluvoxamine (cytochrome P450 inhibitor) markedly increase the levels of melatonin in the blood. Furthermore, the bioavailability of melatonin is also significantly increased when taken with caffeine or vitamin E/C in human subjects. Hence, it is necessary to deeply understand the pharmacokinetics of melatonin in the serum and its interaction with other substances or adjusting the dose in different situations and individuals.

## 4. Neuroprotective Properties of Melatonin against Bacterial Meningitis

### 4.1. The Antibacterial Activity of Melatonin

At present, antibiotic treatment is related to mortality in the early stages of bacterial meningitis. However, antimicrobial resistance is occurring all over the world. In particular, the emergence of multiple antimicrobial resistance makes treatment more difficult. In addition, some antibiotics, such as vancomycin, poorly cross the BBB, which greatly reduces the antimicrobial efficiency. Meanwhile, antibiotics residue in animal products also threatens human health.

Melatonin as an endogenous molecule has been widely investigated in cells and organisms, but few studies are explored in antimicrobial activities of infectious diseases. In 2008, Tekbas and colleagues have shown that melatonin could inhibit the growth of gram-positive and gram-negative bacteria. In the study, it was reported that melatonin play bacteriostasis ability against methicillin-resistant *Staphylococcus aureus*, carbapenem-resistant *Acinetobacter baumannii*, carbapenem-resistant *Pseudomonas aeruginosa*, *Staphylococcus aureus* ATCC 29123, and *Pseudomonas aeruginosa* ATCC 27853. The minimum inhibitory concentration(MIC)concentrations of melatonin was, respectively, 250 μg/mL, 125 μg/mL, 125 μg/mL, 250 μg/mL, and 125 μg/mL at 24 h of incubation. Melatonin’s MIC values were, respectively, decreased to 250 μg/mL, 125 μg/mL, 125 μg/mL, 250 μg/mL, and 125 μg/mL after 48 h of incubation. Moreover, it was found that melatonin in lower doses has a potent antimicrobial function, which is possibly caused by the reduction of intracellular substrates, which makes bacteria enter the death phase earlier [74]. It is necessary to bind free iron for bacterial growth. Melatonin has a high metal-binding capacity, such as iron, and can resist bacterial growth by binding free iron in the cytoplasm. Konar et al. have demonstrated that melatonin at a concentration of 300 μg/mL could effectively inhibit *Candida albicans* by reducing lipid levels. Moreover, it was reported that melatonin can interact with receptors on the neutrophils, and promote neutrophil extracellular trap (NET) formation to enhance the antibacterial ability of neutrophils, then contribute to the clearance of *E. coli* and *S. aureus* in mice to relieve sepsis caused by bacteria [75].

### 4.2. Melatonin and Immune Activation

Usually, the innate immune system is regarded as the first line of defense against invading pathogens. The complement system plays an important role in clearing pathogens, such as complement-mediated phagocytosis and opsonization of inflammation [37]. Similarly, outer membrane protein A of *E. coli* is able to bind to C4bp for resisting the serum bactericidal activity [76]. During bacterial infection and inflammation in CNS, brain resident cells can produce complement factors except for monocytes and macrophages, resulting in recruiting lots of leukocytes and causing the inflammatory storm. In some cases, it was demonstrated that there was a decreased numbers of leukocyte, reduced cytokines, and chemokines in the CSF of C1q and CR3^−/−^ mice compared with WT mice [77]. Recently, numerous experiments in bacterial meningitis have shown that complement intervention, such as complement monoclonal antibody, was beneficial in the treatment of acute bacterial meningitis. Previous studies reported that the change of serum melatonin levels was related to the complement system. Pro-inflammatory factors and complement proteins are associated to promote Aβ deposits in Alzheimer’s disease (AD). In AD mice, melatonin contributes to improve learning and memory by significantly inhibiting the expression of interleukin-1α (IL-1α) and complement 1q in the hippocampus [78]. Meanwhile, serum melatonin is closely related to complement 3 or complement 4 levels in patients with depression, but the detailed mechanism would be deeply explored. These results do demonstrate the proposed regulation of melatonin on complement proteins expression. Unfortunately, there are no associated studies on melatonin regulating complement resistance to bacterial meningitis and more mechanisms.

TLRs of the immune cells recognize different bacterial pathogen-associated molecular patterns (PAMPs), and TLR activation is a key step in the meningeal inflammatory response, prevents bacterial growth, and also participates in meningitis-induced tissue damage [38,39,40]. Among TLRs, TLR2, TLR4, TLR9 are involved in the pathogenesis of bacterial meningitis [79]. TLR2 is mainly activated by lipoteichoic acid and TLR4 interacts with lipopolysaccharides (LPS) or pneumolysin. TLR9 can interact with bacterial DNA. MyD88 signaling molecule is stimulated during activation of TLRs, which is necessary to induce an effective immune response. In clinical tests of blood samples from child patients with bacterial meningitis and healthy adult, Zhang found that TLR2 and TLR9 with polymorphism gene were markedly higher in Chinese children with bacterial meningitis (seizures), and it is suggested that they may be related with severity and prognosis [80]. For pneumococcal meningitis, TLR2 and 4 are central to resist pathogens invasion and regulate the host inflammation. For example, weakened immune response, increased *S. pneumoniae* burden, and low expression of antimicrobial peptides were found in TLR2/4 double knockout mice [81,82,83]. At present, many researchers focus on selecting effective adjuvant treatment with the drug to interfere with the TLR pathway; for instance, activin A can increase phagocytosis of *E. coli* k1 by microglial cells stimulated by TLR2, 4, and 9 agonists without inducing excessive inflammatory response [84]. However, there is no study on exploring the protective mechanisms of melatonin in bacterial meningitis focusing on TLR innate signaling. However, in hepatic ischemia/reperfusion study, mechanisms that melatonin effectively protect the liver by attenuating the increased level of MyD88, TLR3, and TLR4 protein expression have been intensively investigated, and it was also been found that the inhibitory effects of melatonin on the MyD88 signaling pathway of TLR system was related with suppression of activation of NF-κ B, mitogen-activation protein kinase s (MAPKs), which contribute to the pathogenesis of bacterial meningitis processes [85,86,87].

### 4.3. Melatonin and Pro-inflammatory Cytokine

After bacterial infection, there are many resident cells in CNS and invading immune cells from the bloodstream, which can produce pro-inflammatory cytokines to respond to bacterial components and replication. In a study of patients with bacterial meningitis, the levels of pro-inflammatory cytokines were detected in the CSF. IL-6, IL-1β, and tumor necrosis factor-α (TNF-α) are produced by brain microvascular endothelial cells (BMECs), astrocytes, and microglial cells at the early stages of bacterial infection [88]. These early-produced cytokines can increase the expression of some adhesion factors on the BMECs, which recruit a large number of neutrophils into the CSF. Moreover, massive inflammatory reactions induced by pathogens can contribute to functional and structural brain damages, as well as are major features of bacterial meningitis [89]. Lots of pathological reports have shown that the release of increased pro-inflammatory cytokines (e.g., IL-1β, IL-6, TNF-α) in activated microglial cells may promote neuronal apoptosis in the hippocampal regions [90,91]. The excessive release of pro-inflammatory factors, likewise, could break the integrity of BBB and interrupt the bioenergetics activity or the metabolic activity of injured neurons [92].

Within the past years, experts have well demonstrated the anti-inflammatory properties of melatonin in alleviating neuron damage and improving the recovery of injured neurons’ functions [93,94,95]. Melatonin was found to inhibit inflammatory response by decreasing MMP-9 expression and vascular endothelial growth factor expression, thus preventing the disruption of tight junction proteins (Zonula occluding-1 (ZO-1); occluding; claudin-5) and attenuating brain edema following BBB dysfunctions [96]. In adult rats inoculated with acute *Klebsiella pneumonia* meningitis model, it is found that TNF-α, IL-1β, and IL-6 levels were significantly decreased following melatonin dose of 100 mg/kg administration [18]. Then, the study has clearly demonstrated that melatonin treatment can successfully block microglial activation and reduce inflammatory responses in the hippocampal and subsequently rescues hippocampal neurons from apoptotic damage [18,95,97]. However, when melatonin treatment was started after 12 h in a rabbit *Streptococcus pneumonia* or *Escherichia coli* meningitis model, melatonin exerted anti-inflammatory effects but did not alleviate neuronal injury [98]. The reason for such an issue may be associated with the time of melatonin treatment.

### 4.4. Melatonin and MMPs

Over the past years, lots of clinical and animal model reports demonstrated that MMPs play a central role during the development of bacterial meningitis. MMPs as an endopeptidase are involved in cleaving extracellular matrix proteins but also regulating signaling molecules and receptors [99,100,101]. During bacterial infection, resident activated-cells (microglia, astrocytes, and neurons) and blood-derived leukocytes (neutrophils, macrophages, and lymphocytes) can release the MMPs [102,103,104,105,106].

In bacterial meningitis, MMPs are major mediators of BBB damage and modulators of inflammation in the brain, which cleave extracellular matrix and nonmatrix proteins under pathophysiological conditions [107,108,109]. Over past years of clinical and experimental studies, upregulation of MMP-9 in human BM was reported in 19 patients [110]. MMP-8 also is up-regulated in CSF of children with BM [110]. MMP-9 can increase the permeability of the BBB by degrading collagen, proteoglycan, or basal laminin, resulting in pathogen invasion and leukocyte extravasation [111]. Moreover, MMPs can cleave inflammatory cytokines and chemokines and stimulate their production for hyperinflammatory reactions driving brain damage [112,113]. For example, a high level of MMP-9 has been described that can improve the risk for the development of neuronal damage, such as hearing impairment and secondary epilepsy in infected children [114,115]. To date, lots of adjuvants targeting MMPs are applied in clinical studies of bacterial meningitis. In infant rats with pneumococcal meningitis, Trocade as an adjuvant can inhibit collagenases and gelatinase activity, decrease pro-inflammatory factors and mortality, and alleviate CNS injury. Furthermore, antibiotic treatment might increase the expression of MMP-9, and antibiotics with dexamethasone could inhibit the expression of MMP-9 in rats with *Streptococcus pneumonia* [116].

Treatment with melatonin protected the integrity of the BBB and against neuroinflammation by regulating MMP gene expression and activity [117,118]. Under physiological and pathological conditions, TIMP-1 (Tissue inhibitors of metalloproteinase-1) bind to the MMP catalytic domain for inhibiting MMP-9 activity [111]. Moreover, the administration of exogenous melatonin actually increases the TIMP-1 expression by inducing MAPK pathways, which reduces the MMP-9 translation and activity [111]. MMP-9 secretion induced by IL-1β in pericytes can disrupt VE-cadherin, occluding, claudin-5, and ZO-1, resulting in increasing BBB permeability [117]. The melatonin can downregulate MMP-9 and upregulate TIMP-1 gene expression through regulating the NOTCH3/ nuclear factor kappa B (NF-κB)/p65 signaling pathway in pericytes to protect the disruption of the BBB integrity induced by IL-1β [117,119].

In a mouse model of meningitis induced by LPS, melatonin (5 mg/kg) significantly attenuated cerebral MMP-9 activity following brain inflammation; and in the RAW264.7 and BV2 cells, the results showed that pretreatment or cotreatment with melatonin effectively inhibited LPS-induced MMP-9 activation [96]. It has been reported that melatonin control redox-dependent negative regulation of the MMP-2 gene and also can induce MMP-9 downregulation by inhibiting TNF-α [108,120]. Meanwhile, MMPs are involved in the apoptosis and death of neurons, and the injury of hippocampal neurons was alleviated in MMP-9-deficient mice with global ischemia [121]. Based on many reports, melatonin can play an important role in the neuroprotective effect by regulating MMP-9 activation, and melatonin may tightly bind to the active site of MMP-9 for inhibiting MMP-9 activation. Hence, MMP-9 may be a major target of melatonin in neuroprotection against brain injury.

### 4.5. Melatonin and Oxidative Stress

Oxidative stress is the primary cause of brain injury, which includes high lipid content, ROS, and RNS [41,42,122,123]. Under physiological conditions, free radical (ROS, RNS) generation and antioxidant response are usually balanced. However, oxidative stress can be induced if ROS or RNS formation is excessively increased or antioxidant levels are depleted under pathological conditions [124,125]. Studies in experimental animals and humans with bacterial meningitis have shown that neuronal injury and the BBB breakdown are regulated by ROS, RNS, nitric oxide, and peroxynitrite [126,127]. For example, oxidative stress facilitates the disruption of the BBB by reducing the expression of tight junction proteins (claudin-5, occludin, ZO-1, and junction adherensive molecular-1). Oxidative stress can also markedly activate MMPs and break the BBB integrity during pneumococcal meningitis. Leib and colleagues have found that ROS was produced from predominantly polymorphonuclear leukocytes in the subarachnoid and ventricular space, cortical vessels, endothelial cells in group B streptococci meningitis [128,129]. There is also a large generation of ROS from microglia, neurons, and astrocytes induced by *E. coli* lipopolysaccharides, cytokines (TNF-α and interleukin-1β [IL-1] β) [130,131,132,133]. Oxidative stress or free radicals increasingly became a vital event in promoting the development of neuronal injury during bacterial meningitis. For example, peroxynitrite can induce cytotoxicity through inhibition of mitochondrial function, leading to depletion of NAD^+^ and ATP, resulting in neuronal cell death [134,135]. In vivo, phenyl *N*-*t*-butylnitrone (PBN), which is a radical scavenger, prevented CNS injury caused by group B streptococcal meningitis, and NAC as antioxidant can decrease neuronal death induced by pneumococcal meningitis [136]. Moreover, NAC has been applied in clinical treatment for several years with minor side effects.

Melatonin is a highly effective free radical scavenger and powerful antioxidant with direct or indirect effects. The massive production of ROS and RNS causes significant nerve injury. Melatonin has directly nonreceptor-mediated free radical scavenging activity, as well as it eliminates ROS including hydroxyl radical, peroxyl radicals, hydrogen peroxide, and hypochlorous acid [137,138]. Usually, lipid peroxidation is regarded as a marker of oxidative stress [139,140,141]. The lipid peroxidation marker 4-hydroxynonenal and malondialdehyde contribute to superoxide anion (O_2_^−^) production and are elevated in the cerebrospinal fluid (CSF) of patients with pneumococcal meningitis [142,143]. Melatonin can significantly reduce 4-Hydroxynonenal (4-HNE) and malondialdehyde (MDA) concentration; therefore, inhibit lipid peroxidation and oxidative stress against acute tissue injury in a study [144]. Additionally, melatonin likewise inhibits nitric oxide synthase and lipoxygenase [144,145,146,147]. Bacterial meningitis stimulates inducible Nitric Oxide Synthase (iNOS), resulting in significantly increasing NO levels in the brain and induce neurotoxicity [3,148]. Lipid A treatment enhances iNOS expression by activating NF-κB signaling cascades in the choroid plexus epithelium that is a part of the blood–CSF barrier against microbial pathogens and plays a crucial role in brain inflammatory processes in bacterial meningitis [149]. Meanwhile, the inhibition of iNOS expression completely prevented brain damage induced by *E. coli* K1. NO as a major inflammatory mediator is also responsible for the enhanced invasion of *E. coli* K1 into human brain microvascular endothelial cells (HBMECs), which is an in vitro model of the BBB [150]. Melatonin directly reduces nitrite concentration that represents the level of iNOS expression in microglial cells and the CSF of rabbits infected with *Streptococcus pneumonia*, resulting in reducing neuronal injury [151,152].

Furthermore, melatonin can play an indirect role in antioxidants by inducing antioxidative enzyme expression [153,154]. Within the past years, several animal studies on melatonin have shown that melatonin can stimulate lots of antioxidative enzymes including superoxide dismutase (SOD), MnSOD, CuZnSOD, glutathione peroxidase, glutathione reductase, and catalase [145,155]. For example, SOD can catalyze the breaking down of superoxide in H_2_O_2_ and oxygen molecule, and CAT is able to exert detoxification H_2_O_2_ [145]. The activity of SOD in meningitis patients is related to ROS formation due to lipid peroxidation for neutralizing the free radical [41]. SOD mimetics pre-treatment could alleviate brain edema and decrease intracranial pressure and CSF leukocyte count in a bacterial meningitis rat model [156]. In melatonin-treated rabbits infected with *Streptococcus pneumonia* study, melatonin significantly increases the activity of SOD and reduces the nitrite concentrations for resisting oxidative stress [98]. The levels of Glutathione in CSF of patients with meningitis were significantly reduced, which enhance the risk of oxidative stress and lead to severe neurological dysfunction [98,157,158]. Melatonin can increase intracellular GSH levels by stimulating γ-glutamylcysteine synthase to protect the nervous system from oxidative damage [159].

### 4.6. Melatonin and Mitochondrial Dysfunction

It was demonstrated in several studies that mitochondrial dysfunction has a related function in the pathogenesis of bacterial meningitis [127,160,161]. Normally, the mitochondrion plays an important role in aerobic metabolism for providing energy and maintaining cellular homeostasis [162,163]. It is well known that neurons of the brain have a high metabolic rate and contain a large number of mitochondria, therefore, more susceptible to the reduction of energy metabolism [126]. Lots of brain biological processes are regulated by mitochondria, including ATP production, oxidative stress, calcium balance, and apoptosis [161]. Approximately 50% of patients with bacterial meningitis in the epidemiological investigation have demonstrated that cerebral oxidative metabolism was affected, indicating mitochondrial dysfunction [127]. For example, in patients with severe streptococcus meningitis, the study recorded the data that can reflect cerebral cytoplasmic redox state by evaluating cerebral interstitial lactate/pyruvate (LP) ratio, and increase in LP ratio indicates impaired cerebral oxidative metabolism, which is determined by mitochondrial dysfunction [126]. Then, the cell energy is obtained by oxidative phosphorylation that depends on various respiratory enzyme complexes located in the inner mitochondrial membrane [126]. The experimental studies have demonstrated that pneumococcal meningitis can inhibit mitochondrial chain complex I in the brain, causing impairment of energy metabolism for facilitating the development of pathogenesis [164]. In fact, excess ROS production in bacterial meningitis can induce defects in the mitochondrial chain, causing impairment in oxidative phosphorylation that promotes ATP generation and more ROS [165]. More and more ROS can result in mitochondrial dysfunction, then cause the release of apoptosis-inducing factors into the cytosol; these factors have been found that execute the caspase-independent pathway [166]. Furthermore, numbers of polymorphonuclear leukocytes in animal models of pneumococcal meningitis can also increase the release of pro-apoptotic factors such as cytochrome c from mitochondria into the cytosol, which leads to caspase-3 cleavage, resulting in neuronal apoptosis [167,168]. Hence, compounds or drugs that can maintain the mitochondrial function and inhibit associated apoptotic signaling pathways will be effectively used in combating bacterial meningitis.

Under pathological conditions, mitochondria are regarded as an important target of melatonin due to the accumulation of melatonin in high concentrations on mitochondria [169]. Melatonin can relieve mitochondrial dysfunction by scavenging free radicals, regulating the electron transport chain, and increasing antioxidase activities. In in vivo or in vitro experiments, it has been found that melatonin can attenuate mitochondrial dysfunction in sepsis and protects mitochondria from oxidative damage by scavenging free radical [170]. Melatonin also plays a critical role in protecting mitochondria by increasing the activity electron transport chain, improving ATP production, attenuating calcium overload, inhibiting ER stress, regulating mitochondrial gene expression, and preventing mitochondrial apoptosis [171]. The study has been shown that melatonin can interact with complexes I and IV of the mitochondrial electron transport chain to promote electron flux under the normal physiological conditions for increasing ATP production for maintaining mitochondrial homeostasis [172,173,174]. In addition, melatonin increases GSH synthesis for improving the mitochondrial defense mechanism [175]. At the same time, melatonin can increase the activity of NADH dehydrogenase in brain mitochondria against neurotoxicants [176,177]. The protective function of melatonin against apoptosis has been demonstrated in a number of neural injury studies [166]. It has been reported that melatonin diminishes apoptosis by increasing anti-apoptotic proteins, such as B-cell lymphoma-2 (Bcl-2), or inhibiting pro-apoptotic proteins, such as Bax [178]. For instance, pre-treatment of melatonin can induce the overexpression of Bcl-2 and inhibit caspase 3 or Cyt c release under oxidative stress [178]. Melatonin also facilitates Bax to translocate into the mitochondria, leading to reducing the apoptotic tendency [178]. However, there are few studies on melatonin in alleviating mitochondrial damage in bacterial meningitis. Thus, according to multiple mechanisms or functions of melatonin, it is worth exploring to protect mitochondria from dysfunction induced by oxidative stress or other virulence.

### 4.7. Melatonin and Signaling Pathways

Many studies have shown that there were some major intracellular signaling pathways involved in the process of bacterial meningitis, such as nuclear factor kappa B (NF-κB) pathway, phosphoinositide 3-kinase (PI3K)/Akt pathway, mitogen-activation protein kinase (MAPK) pathway. These signaling pathways contribute to developing the process of bacterial meningitis. For instance, once the pathogens invade the BBB, most bacteria are able to activate the NF-κB pathway by phosphorylation of serine residues on the IκB proteins, resulting in increasing inflammatory factors, chemokines, bacterial invasion of BMECs, and polymorphonuclear (PMN) migration across the BBB. For example, IbeA protein of *E. coli* K1 interacted with vimentin of BMEC and stimulates NF-κB and extracellular signal-related kinases 1/2 (ERK1/2) activation, resulting in promoting bacterial invasion and PMN transmigration across the BBB [179,180]. Meanwhile, NF-κB pathway and PI3K/Akt/mammalian target of rapamycin (mTOR) signaling pathway were reported that involved in inhibiting autophagy for increasing intracellular bacterial survival rate in *E. coli* K1 meningitis [181]. *Streptococcus suis* serotype 2 (SS2) can interact with epidermal growth factor receptor to initiate MAPK-ERK1/2 and NF-κB pathway in hBMEC that facilitate the proinflammatory cytokines and chemokines expression [182].

This evidence expands our ideas on finding a drug to prevent or treat bacterial meningitis. In past years, brain-derived neurotrophic factor (BDNF) was demonstrated that played an important role in anti-inflammatory and anti-apoptotic in CNS diseases. In the rat model of pneumococcal meningitis, BDNF supplement can effectively reduce inflammation and hippocampal apoptosis by regulating NF-κB pathway and PI3K/Akt/mTOR signaling pathway [181]. Signaling pathway inhibitors have been used in bacterial meningitis, such as U0126 (MAPK inhibitor), CAY10657, or BAY-11072 (NF-κB inhibitor), which could effectively inhibit neuroinflammation in vitro.

In previous reports, the MyD88/NF-κ B signaling pathway could cause neurological injury in bacterial meningitis and melatonin inhibits NF-κ B-driven signaling for protective and anti-inflammatory action in the LPS-stimulated RAW 264.7 and BV2 cells [96]. In addition, the inhibitory effect of melatonin on post-inflammatory NF-κ B translocation and proMMP9 activation is effective following LPS-induced meningitis [183]. Similarly, some studies have demonstrated that the PI3K/Akt signaling pathway is important in alleviating neuronal apoptosis and promoting neuronal survival [184]. Additionally, Melatonin can inhibit neuron apoptosis and increase cellular survival. In mice experiments, melatonin treatment reduced p53 phosphorylation by the PI3K/Akt pathway for decreasing apoptosis in the brain [85]. Meanwhile, melatonin regulates the expression of brain and muscle Arnt-like protein 1 (Bmal 1) by PI3K/Akt pathway and increases cellular survival via survival kinases in vivo and in vitro [185]. Hence, melatonin can be regarded as a novel strategy targeting the major signaling pathway, for the prevention and treatment of bacterial meningitis.

### 4.8. Other Functions of Melatonin in Bacterial Meningitis

Bacterial adhesion is a prerequisite for the development of infection and usually interacts with host-specific surface adhesion receptors for nutrient intake, promoting bacterial invasion and immune evasion. There are two natural barriers to defense against meningitis bacteria before they invade the CNS, respectively, the mucosal barrier and the BBB. Meningitis bacteria can use adhesion or other bacterial virulence factors to bind to surface receptors of barriers for invading into the CNS. For example, type IV pili can contribute *N. meningitides* to adhere to the BBB by targeting CD147 receptors on BMEC [186]. In our study, we have been found OmpA and IbeA in APEC TW-XM (isolated from duck) could, respectively, induce gp96 and caspr1 receptor expression, as well as contribute to bacterial adhesion and disrupt the integrity of the BBB via activating the focal adhesion kinase (FAK) pathway. Then, we found that melatonin can decrease the expression of OmpA and IbeA, resulting in reducing the adhesion and invasion of APEC TW-XM (unpublished data). Lots of scholars focus on exploring the mechanisms of bacteria-binding to host receptors during bacterial infection. However, few studies have explored the mechanisms of how melatonin affects the interaction of meningitis bacteria and host receptors. Hence, it is might be a new and useful target with a broad spectrum for the prevention or treatment of meningitis bacteria.

The gut microbiome as a line provides resistance against foreign pathogens. Commensal microbes in the gut can release bacteriocins, utilize nutrient depletion mechanisms, regulate metabolism and immunity to resist pathogens. In infectious diseases, many studies have shown that pathogens and induced cytokines caused gut dysbiosis, resulting in the promotion of colonization of pathogens. Moreover, antibiotic treatment of immunocompromised host would enhance susceptibility to bacterial meningitis, and it also have been demonstrated to mediate microbiota damage. It was reported that commensal bacteria can decrease the bacterial adhesion in *Listeria monocytogenes* meningitis. Particularly, the clostridiales of commensal bacteria exerted antibacterial activity in vitro and conferred into germ-free mice to increase resistance against *L. monocytogenes*. These studies indicated that the intestinal microbiome is closely related to the disease process [187].

Several works suggest that melatonin can regulate gut microbiome balance and relieve some diseases. Ren and colleague found that melatonin supplementation could alleviate weanling stress and decrease intestinal ETEC infection by shaping the composition of intestinal microbiota in weanling mice. Meanwhile, this study also demonstrated that melatonin failed to alleviate weanling stress and defense ETEC infection both in antibiotic-treated weanling mice and germ-free weanling mice [14]. It is suggested that melatonin could regulate the gut microbiome to alleviate disease. In the spinal cord injury (SCI) mice model, gastrointestinal system dysfunction is a typical symptom, and alteration of the gut microbiome may affect disease progression. It has been demonstrated that melatonin treatment can not only improve some main pathology of SCI but also regulate the composition of intestinal microbiota (including increase in abundance of *Lactobacillus* and *Lactobacillales* and decrease in the abundance of *Clostiddiales*). The neuroprotective effect of melatonin on SCI was significantly reduced in gut dysbiosis mice model induced by antibiotics treatment [188]. At present, there are few studies to explore the function of melatonin on the prevention and treatment of bacterial meningitis by alteration of intestinal microbes. In our study, we have found that APEC TW-XM can induce gut dysbiosis and melatonin could prevent APEC TW-XM-induced bacterial meningitis by maintaining gut microbiome in Institute of Cancer Research (ICR) mice. We applied melatonin by intraperitoneal injection and found that melatonin can maintain gut microbiome homeostasis by increasing abundance of *Alistipes*, *Parabacteroides,* and *Lactobacillus*, as well as decreasing in the abundance of *Strenotrophomonas*, yet lost the function of prevention in antibiotic-treatment ICR mice (unpublished data). Hence, intestinal microorganisms can be regarded as a target of melatonin. Melatonin could regulate host metabolism by improving gut dysbiosis, so as to enhance the resistance to pathogens or alleviate nerve injury in bacterial meningitis.

## 5. Conclusions and Future Perspective

The outcome of bacterial meningitis is related to the destruction of the BBB integrity, excessive inflammatory responses, and nerve cell apoptosis. Although advances in antibiotic therapy and vaccine development, bacterial meningitis still remarkably causes high morbidity and mortality among children, infants, elders, and immunocompromised patients. The most difficult to prevent and treat bacterial meningitis is the diversity of pathogens and severe nerve injury. However, the limitation of vaccine and antibiotic resistance increases more difficulty in preventing and treating bacterial meningitis, and cannot timely and effectively prevent neural tissue from injury. Another major hurdle for bacterial meningitis treatment is the inefficient delivery of some antibiotic or macromolecular drugs into the brain due to the BBB.

It is clearly demonstrated that melatonin plays a beneficial role in neurological diseases. These functions mainly depend on the chemical and biological characteristics of melatonin. In recent years, most of the studies pointed out that melatonin with high solubility, which mainly releases from the pineal gland and across the BBB, is a functionally diverse molecule involved in the regulation of physiology, modulation of the immune system, and neuroprotection function [189,190]. In this review, we have shown that melatonin plays an important role as antibacterial, antioxidant, free radical scavenger, and immune system regulator, and so on. Furthermore, there are many clinical studies of melatonin on neuroprotection in different neurological diseases. For example, it was demonstrated that a 20 mg dose of melatonin supplement could reduce inflammation in serum and increase survival of newborns with sepsis [191]. In addition, the application of melatonin at a dose of 10 mg/kg, 5 times a day, could reduce newborns’ new epilepsy and brain anomalies. In multiple sclerosis patients, supplement melatonin at 5 mg/day could improve the life quality of 102 patients by reducing MDA. Hence, these studies provide a positive effect on the widespread use of melatonin [192].

Until now, there is no clinical trial of melatonin on the treatment or prevention of human meningitis. The findings on CSF of patients with meningitis have shown that *N*^1^-acetyl-*N*^2^-formyl-5-methoxykynuramine (AFMK) levels in the presence of the melatonin metabolite were in a remarkably high concentration, aimed to control the intensity of the inflammatory process by scavenging ROS [71]. Thus, it is well considered that the increment in AFMK concentration from melatonin metabolite in meningitis may be a physiological response to protect the brain tissue damage [71]. In in vivo or in vitro experiments, the ability of melatonin and its metabolites to cross the BBB into CNS has been identified for protecting nerve cells from injury and inducing neuritogenesis [98]. Even if there are no harmful effects of melatonin at different doses on the rodent meningitis model at present, the effective dose of melatonin for neuroprotection may be different due to bacterial meningitis caused by different pathogens. In a rabbit *Streptococcus pneumoniae* meningitis model, melatonin as an adjunctive treatment at a dose of 1.67 mg/kg 12 h after infection had anti-inflammatory effects but did not alleviate neuronal injury. Moreover, in the rat *Klebsiella pneumonia* meningitis model, melatonin effectively reduced inflammatory response and decreased microglial activation and the number of apoptotic neurons at dose 100 mg/kg [18]. According to the patient’s age, autoimmunity, and bacterial type, we need to consider the effective dose of melatonin supplement, which stage to supplement, route of melatonin, melatonin supplement time, and the safety of melatonin in future clinic trials of bacterial meningitis. The aim is to make melatonin play a greater role in bacterial meningitis. Thus, the safety and effective treatment methods of melatonin for preventing or treating bacterial meningitis patients need more clinical studies.

**Figure 2:**
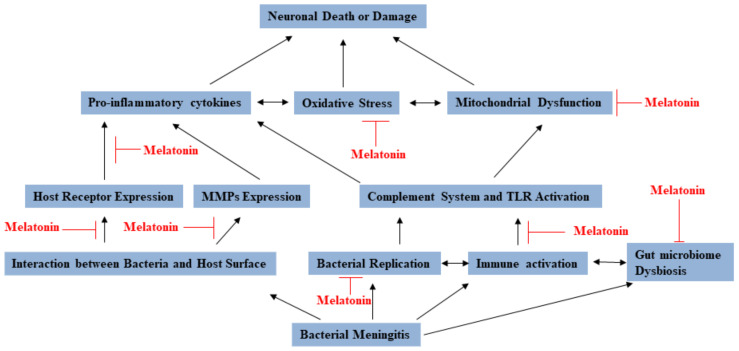
The multiple functions of melatonin that provide prevention and protection against bacterial meningitis.

In conclusion, melatonin has been found to have various mechanisms against bacterial meningitis (see Figure 2). Lots of reports identified that melatonin seems to be very promising, but there are still more studies on discussing and establishing guidelines to the clinical application of melatonin for preventing or treating bacterial meningitis patients.

## Figures and Tables

**Table 1:** The treatments of bacterial meningitis.

Main Meningitis Bacteria.	Mainly Infected Age Group	Vaccine	Antibiotic	Adjunctive Treatment	Reference
*Streptococcus* *pneumoniae*	Children < 5 years; Adults > 50 years	Live attenuated vaccine (Whole-cell vaccine); Inactivated vaccine (Whole-cell vaccine); Subunit vaccine: Polysaccharide vaccine (PPV23), Conjugate vaccine (PCV7/10/13/15), Protein-based vaccine (PcsB, StkP, PsaA, PspA, PcpA, PhtD, PlyD1, Ply).	Penicillin; Macrolides.	Magnesium; Efflux pump inhibitors; C5 antibodies; Dexamethasone; Corticosteroids.	[44,45,46,47]
*Neisseria* *meningitis*	Children < 5 years Adolescents	Conjugate vaccine (MenACWY, Hib_MenCY-TT, Men A conjugate vaccine, Men C conjugate vaccine); Polysaccharide vaccine (MPSV4); Protein-based vaccine (Multicomponent Men B vaccine, Men B bivalent vaccine)	Penicillin; Ceftriaxone; Ciprofloxacin; Rifampicin.	BB-94 (MMP inhibitor); Doxycycline;	[48,49,50]
Group B *Streptococcus*	<3 months	CPS conjugate vaccines (CPS-CRM_197_ GBS conjugate vaccine); Protein-based GBS vaccines (Alpha-like protein, Rib, AlpC); Polysaccharide conjugates vaccine (serotypes Ⅰa, Ⅰb, and Ⅲ)	Penicillin G; Clindamycin; Erythromycin; Fluoroquinolones; Ampicillin; First-, second-, and third-generation cephalosporins; Carbapenems; Vancomycin.	Gentamicin; Migration inhibitory factor inhibitor (ISO-1); Insulin; MAPK inhibitors; Brain-derived neurotrophic factors; Hypothermia.	[51,52,53,54,55,56,57,58]
*Streptococcus suis*	Adults	Autogenous bacterins; Subunit vaccine (muraminidase-released protein, suilysin, extracellular factor); 6-phosphogluconate-dehydrogenase; SsnA (the cell wall-associated DNase); Subtilisins; Glycoconjugates; Capsular material coupled with botulinum toxin.	Penicillin G; Ceftiofur; Amoxicillin; Gentamicin; Florfenicol; Fluoroquinolones	Aluminum hydroxide adjuvant; Imugen^®^; Rehydragel^®^ and Emulsigen^®^.	[59,60,61]
*Escherichia coli* K1	<3 months	Mutation of aro A gene; Recombinant ISS gene; Outer membrane protein A (OmpA_TM_, transmembrane domain; OmpA_per_, periplasmic domain; OmpAVac); Capsular polysaccharides	Gentamicin; Ceftriaxone; Penicillin G; Ampicillin; Amoxicilline; Meropenem.	Pentoxifylline; Palmitoylethanolamide;	[62,63,64,65,66,67]

## Data Availability

Not applicable.

## Abbreviations

BM	Bacterial meningitis
CNS	Central nervous system
BBB	Blood-brain barrier
GBS	Group B Streptococcus
HIV	Human immunodeficiency virus
CM	Cryptococcal meningitis
HiB	Haemophilus influenzae
MIC	Minimum inhibitory concentration
TNF-α	Tumor necrosis factor-α
TLR	Toll-like receptors
IL-1α	Interleukin-1α
NLRs	Nod-like receptors
LPS	Lipopolysaccharides
PAMPs	Pathogen-associated molecular patterns
NF-κ B	Nuclear factor kappa B
MAPK	Mitogen-activation protein kinase
PBN	Phenyl *N*-*t*-butylnitrone
4-HNE	4-Hydroxynonenal
MDA	Malondialdehyde
iNOS	inducible Nitric Oxide Synthase
HBMEC	Human Brain Microvascular Endothelial Cell
IL-6	Interleukin-6
ROS	Reactive oxygen species
RNS	Reactive nitrogen species
MMPs	Matrix metalloproteinases
CSF	Cerebrospinal fluid
BMECs	Brain microvascular endothelial cells
AJs	Adherens junctions
TJs	Tight junctions
ZO-1	Zonula occluding-1
CSF	Cerebrospinal fluid
SOD	Superoxide dismutase
PMN	Polymorphonuclear
mTOR	Mammalian target of rapamycin
Bmal 1	Brain and muscle Arnt-like protein 1
FAK	Focal Adhesion Kinase
LP	Lactate/pyruvate
MyD88	Myeloid differentiation factor 88
ERK1/2	Extracellular signal-related kinases 1/2
EGFR	Epidermal growth factor receptor
AFMK	*N*^1^-acetyl-*N*^2^-formyl-5-methoxykynuramine
PCV	Pneumococcal conjugate vaccines
PPV	Pneumococcal polysaccharide vaccine
PspA	Pneumococcal surface protein
Ply	Pneumolysin
